# Inverted ILM Flap Technique in Optic Disc Pit Maculopathy

**DOI:** 10.18502/jovr.v18i2.13189

**Published:** 2023-04-19

**Authors:** Ali Tavallali, Yasaman Sadeghi, Seyed-Hossein Abtahi, Hosein Nouri, Sanam Samadikhadem, Mitra Rezaei, Mehdi Mazloumi

**Affiliations:** ^1^Private Practice, Shiraz, Iran; ^2^Bloomberg School of Public Health, Johns Hopkins University, Baltimore, MD, USA; ^3^Ophthalmic Research Center, Research Institute for Ophthalmology and Vision Science, Shahid Beheshti University of Medical Sciences, Tehran, Iran; ^4^Labbafinejad Medical Center, Shahid Beheshti University of Medical Sciences, Tehran, Iran; ^5^School of Medicine, Isfahan University of Medical Sciences, Isfahan, Iran; ^6^Department of Ophthalmology, Imam Hossein Medical Center, Shahid Beheshti University of Medical Sciences, Tehran, Iran; ^7^School of Medicine, Shahid Beheshti University of Medical Sciences, Tehran, Iran; ^8^Eye Research Center, Rasoul Akram Hospital, Iran University of Medical Sciences, Tehran, Iran

**Keywords:** Flap, Internal Limiting Membrane, Macular Schisis, Optic Disc Pit Maculopathy, Optical Coherence Tomography, Serous Macular Detachment

## Abstract

**Purpose:**

To present the outcome of optic disc pit maculopathy (ODPM) managed successfully with an inverted internal limiting membrane (ILM) flap over the optic disc. A narrative review of ODPM pathogenesis and surgical management techniques are also provided.

**Case Report:**

This prospective interventional case series included three eyes of three adult patients (25–39 years old) with unilateral ODPM and a mean duration of unilaterally decreased visual acuity of 7.33 ± 2.40 months (4–12 months). The pars plana vitrectomy with posterior vitreous detachment induction was performed on eyes, followed by an inverted ILM flap insertion over the optic disc and gas tamponade. Patients were followed for 7–16 weeks postoperatively; best-corrected visual acuity (BCVA) improved dramatically in one patient from 2/200 to 20/25. BCVA in other patients improved two and three lines – to 20/50 and 20/30, respectively. A significant anatomical improvement was achieved in all three eyes, and no complication was detected throughout the follow-up period.

**Conclusion:**

Vitrectomy with inverted ILM flap insertion over the optic disc is safe and can yield favorable anatomical improvement in patients with ODPM.

##  INTRODUCTION

The optic disc pit (ODP) is a rare excavated anomaly of the optic nerve head (ONH), with an estimated prevalence of 2/10,000 and no gender or racial predilection.^[1]^ ODP is most frequently found at the temporal or inferotemporal sections of the ONH, and is assumed as an atypical coloboma of the ONH with dysplastic primitive retinal tissue herniating posteriorly into the subarachnoid space through a defective lamina cribrosa.^[2]^ Mostly, non-complicated ODP are found to be asymptomatic; however, in 25–75% of cases it may develop maculopathy, characterized by cystoid macular changes and/or serous macular detachment.^[3]^ The optic disc pit maculopathy (ODPM) occurs unpredictably and typically in early adulthood, leading to severe visual deterioration, especially in long-standing cases due to the lamellar/full-thickness macular hole development, retinal pigment epithelium (RPE) atrophy, and cystic macular degeneration.^[4]^ Therefore, timely diagnosis and appropriate management of ODPM is necessary. However, given its rarity and controversial pathophysiological aspects, no single treatment is widely accepted and agreed upon as yet.^[3]^ Management strategies mainly include pars plana vitrectomy (PPV) and posterior vitreous detachment (PVD) induction, with or without peripapillary barrier laser photocoagulation, gas tamponade, and internal limiting membrane (ILM) peeling. Recently, covering the ODP using ILM or scleral flaps has been introduced.^[4]^

Here, we describe three cases of ODPM successfully managed with PPV, PVD induction, insertion of inverted autologous ILM onto the ONH (reverse ILM-flap technique), and gas tamponade with non-expansile concentration (14%) of C3F8 by a single surgeon (AT). We also discussed the current understanding of ODPM pathophysiology and reviewed different treatment strategies described in the literature.

##  CASE REPORT

### Case 1

A 39-year-old white woman was presented with the complaint of progressive decrease of vision in her left eye, which had evolved over the prior six months of presentation. Her best-corrected visual acuity (BCVA) was 20/20 and 2/200 in her right and left eyes, respectively. Funduscopy of the right eye was unremarkable, whereas an ODP at the inferotemporal aspect of the optic disc and cystoid macular edema were revealed in the left eye. An OCT of the left eye showed large, schisis-like cystoid spaces in inner and outer retinal layers, with a large intraretinal cyst underneath a very thin overlying fovea. CMT was 685 µm per structural optical coherence tomography (OCT) scan (Spectralis, Heidelberg Engineering, Inc., Heidelberg, Germany).

**Figure 1:**
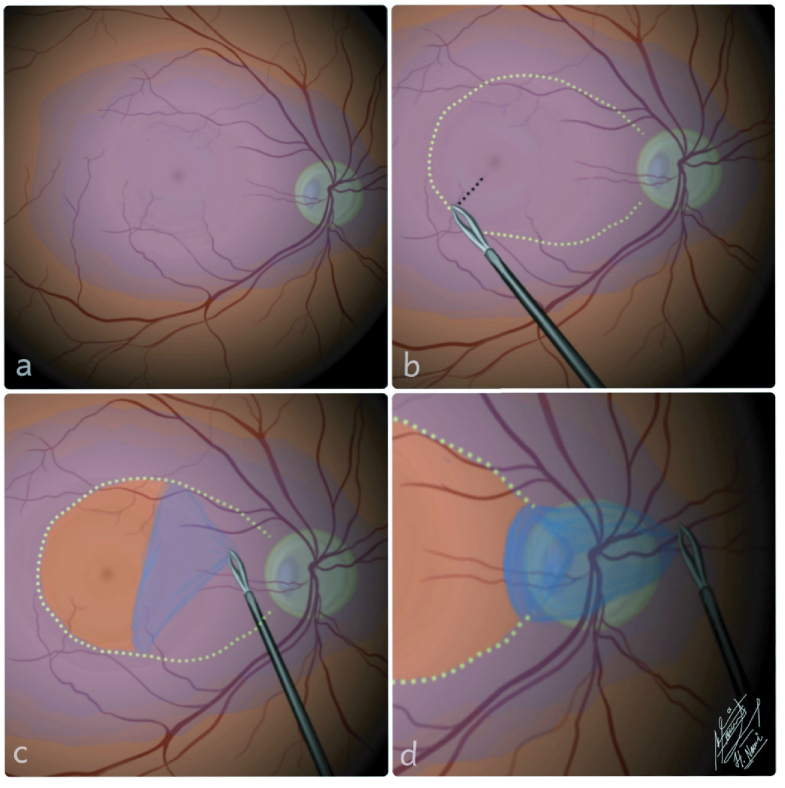
Schematic illustration of the operational technique performed in this study. (a)Membrane-Blue-Dual dye staining was done for internal limiting membrane (ILM) visualization. (b) ILM peeling forceps were used to pinch the ILM at an appropriate position to start peeling, 1 mm temporal to the fovea (black dash line). (c & d) A large ILM flap was peeled up to its pedicles in the optic nerve head adjacency and flattened over the optic disc to cover the pit.

**Figure 2:**
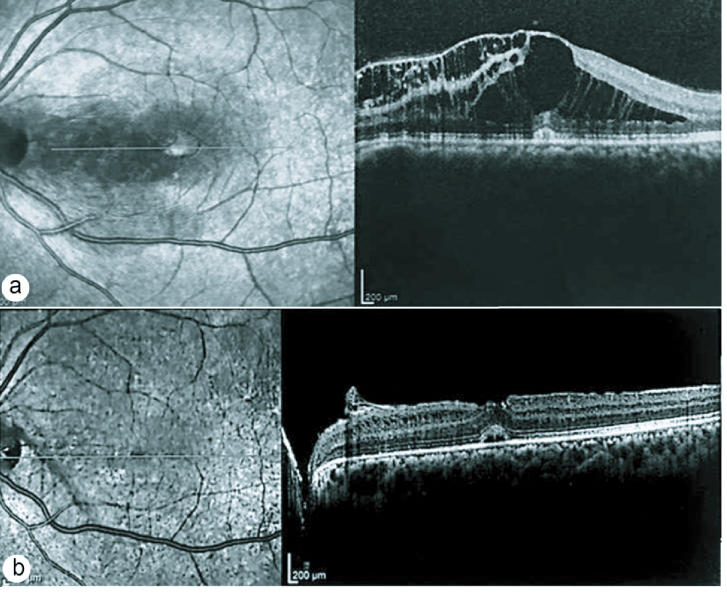
OCT findings in case no 1. (a)Before surgery; schisis-like cystoid spaces in the inner and outer retinal layers and an ample cystoid space in the inner fovea. (b) Twenty weeks postoperatively, ILM remnants appear at the disc margin. Minimal Schises in the inner nuclear layer are noted, and the macula is reattached with small residual sub-foveal fluid.

Under general anesthesia, a three-port, 23-gauge transconjunctival PPV was performed, followed by induction of PVD. As illustrated in Figure 1, ILM was visualized by membrane-blue-dual dye staining (DORC International, EEC) [Figure 1a]. Then, using a 25gauge+ ILM forceps (Grieshaber advanced DSP tip ILM forceps, Alcon Grieshaber AG, Switzerland), a large ILM flap was peeled from 1 mm temporal to the fovea up to its pedicle at 0.2 mm temporal edge of the ONH [Figures 1b & 1c]. The peeled ILM was then inverted and flattened on the optic disc, covering the pit [Figure 1d]. Finally, fluid-air exchange was performed, and C3F8 14% was injected to stabilize the ILM flap over the ODP. The patient was instructed to remain in a prone position postoperatively. At one-month post-operation follow-up, the BCVA of her left eye was 20/40. Two months after the surgery, BCVA had improved to 20/25. Eventually, intraretinal fluid (IRF) was resolved with only a small amount of residual subfoveal fluid. Remnants of ILM were observed at the disc margin. The mean CMT was reduced to 247 µm. The pre- and postoperative OCT scans of this case are presented in [Figure 2a], and [Figure 2b], respectively.

**Figure 3:**
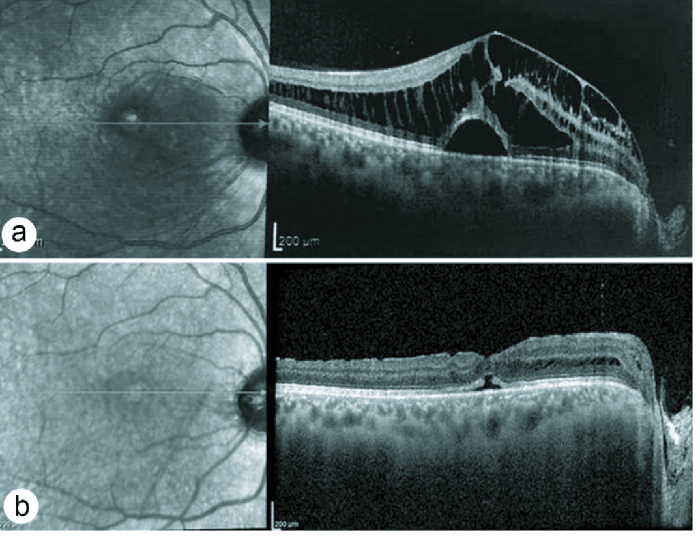
OCT findings in case no 2. (a)Before surgery; note the multilayer inner retinal schisis nasal to the fovea, considerable edema of the Henle's layer, and serous detachment of the macula. (b) Seven weeks post-surgery: Minimal residual fluid beneath the fovea and in the Henle's layer.

**Figure 4:**
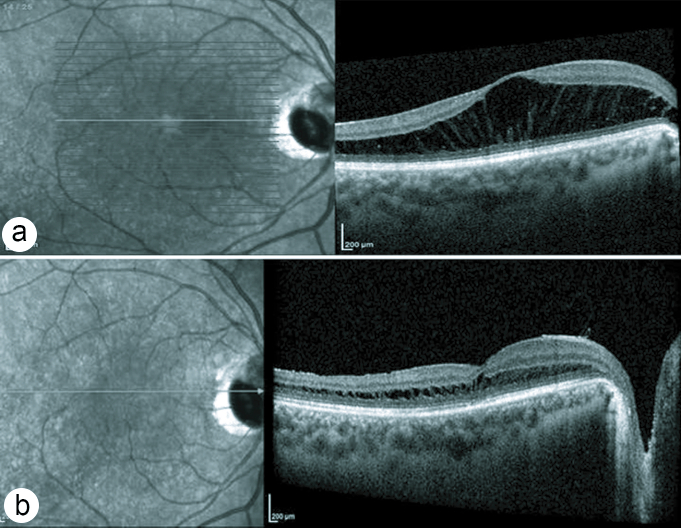
OCT findings in case no 3. (a)Before surgery; huge schisis cavities are visible in the Henle's layer. (b) Sixteen weeks post-surgery: Small residual schisis cavities are left.

### Case 2

A 27-year-old White man was presented with a one-year history of decreased vision in his right eye; his medical history was otherwise unremarkable. Upon examination, his BCVA was 20/100 in the right eye and 20/20 in the left eye. Funduscopic examination of the left eye was normal; a laterally located ODP and associated macular detachment were noted on the right eye funduscopy. OCT scans of the right eye showed a very high mean CMT of 912 µm, multilayer inner retinal schisis nasal to the fovea, marked IRF in the Henle's layer, and serous neurosensory detachment of the fovea [Figure 3a]. The patient underwent the same procedure as the first case. At the seventh-week follow-up, the macular detachment collapsed, the mean CMT reduced to 367 µm, and significant anatomic restoration was achieved [Figure 3b]; BCVA improved to 20/50.

### Case 3

A healthy, White, 25-year-old woman was presented with a history of painless, progressive reduction of central vision in her right eye for four months prior to presentation. The BCVA was 20/70 in the right and 20/20 in the left eye. Funduscopy of the right eye revealed a large inferotemporal ODP and macular edema. In the OCT scan of the right eye, large IRF in the Henle's layer was observed, similar to Case No. 2; the mean CMT was 957 µm [Figure 4a]. The same procedure as described for previous cases was undertaken. Sixteen weeks post operation, her right eye BCVA was 20/30, and retinal edema had significantly resolved. The mean CMT reduced to 330 µm [Figure 4b].

##  DISCUSSION

Over time, the BCVA of eyes with ODPM can deteriorate to 20/200 or worse in many cases. Notably, spontaneous remission is reported in about 25% of cases.^[5,6]^ With spontaneous remission, however, cystic changes of RPE and neurosensory retina and lamellar or full-thickness macular holes are likely to develop, with the risk of permanent visual loss;^[5]^ serous macular detachment may also recur.^[7]^ Even in pediatric cases, whose chances for spontaneous resolution are higher,^[8]^ postponing the surgical interventions to monitor the natural course of maculopathy carries the risk of amblyopia development.^[9]^ Hence, primary surgical management of ODPM is recommended, especially in cases with subretinal fluid (SRF) accumulation.^[1]^

### Pathology and Pathogenesis in the Literature

Historically, vitreous has been suggested as the primary source of fluid leakage into the retina through a thin porous membrane constituted of dysplastic retinal tissue covering the ODP.^[10]^ Through serial histopathological sections of electron microscopy, Christoforidis et al could visualize holes in the diaphanous membrane overlying the ODP bridging with a schisis-like cavity in the retina.^[11]^ SRF drainage through pores in the ODP roof was successfully performed by Johnson and Johnson^[12]^ and Postel et al.^[13]^ One hypothesis is that the negative pressure created by posterior hyaloid traction over the porous ODP-covering membrane generates an inward gradient for movement of the liquified vitreous into the retina.^[11,14]^

Although inconsistent, vitreous strands over the ONH and peripapillary retina have been detected on OCT scans and electron photomicrographs of patients with ODPM.^[2,14]^ The role of vitreous traction in ODPM pathogenesis is amply supported by encouraging remission of maculopathy following vitrectomy^[15]^ or spontaneous PVD^[16]^. Strong vitreoretinal attachment at the disk margin of eyes with ODPM has been reported during surgically induced vitreous detachment.^[7]^ In addition to the role of posterior hyaloid traction, the tangential traction on the retina caused by ILM is also proposed to exert elevational traction on the retina, maintaining the inward fluid gradient into the retina (via ODP).^[17]^ Post-vitrectomy subretinal migration of gas and silicone oil further supports communication between vitreous cavity and subretinal space through ODP; curiously, it happens when no vitreoretinal traction exists, indicating the involvement of other pathogenic mechanisms.^[12]^

Cerebrospinal fluid (CSF) oozing from the adjacent subarachnoid space is the second plausible fluid source; it was first proposed in 1964^[18]^ after Regenbogen et al noticed a pulsating transparent membrane over the ODP during surgery, which they attributed to CSF pressure fluctuations. Akiba et al had a similar observation, which they ascribed to intravitreal traction caused by anomalous Cloquet's canal.^[19]^ Friberg et al reported free pulsation of glial and vitreous remnants overlying ONH into and out of ODP in an eye with visible PVD.^[20]^ They also recognized a retrobulbar cyst communicating with the vitreous cavity through B-scan ultrasonography and documented a constant relative hypotony in the affected eye, presumably due to intraocular fluid drainage into the cyst and, ultimately, the subarachnoid space.^[20]^ In 2006, intracranial migration of silicone oil was noted on brain magnetic resonance imaging scans of a patient with ODPM presented with a headache after vitrectomy and silicone oil injection.^[21]^ The direct communication of intraretinal cystoid spaces and the lamina cribrosa gap in the ODP^[22]^ and their connectivity with the vitreous cavity was later visualized using high-resolution, enhanced depth imaging (EDI), and swept-source (SS) OCT scans. ${}^{\left[23--25\right]}$ As theorized by Johnson and Johnson,^[12]^ anomalous interconnection and fluctuation of the pressure gradient between intra- and extra-ocular spaces enable CSF and vitreous aqua to move through the ODP. When intracranial pressure (ICP) decreases, the syneretic vitreous is sucked toward the communicating perineural space, and then with a rise in pressure, trapped vitreous and/or CSF is ejected into/under the adjacent retina and posterior vitreous cavity. The same mechanism could explain subretinal migration of gas and oil, post-vitrectomy, and the occurrence of maculopathy in pediatric ages when liquified vitreous is uncommon.^[12]^

Regarding the morphology and progression of ODPM, Lincoff et al^[26]^ first introduced the so-called bilaminar retinoschisis concept, in which liquid accumulation emanates from the inner neurosensory retina, extending outward through outer layer lamellar holes. They proposed that true serous macular detachment occurs merely as a complication of longstanding intraretinal edema. Although this view has been widely accepted,^[23]^ it has been challenged by occasional observations of an outer layer hole in OCT scans^[27,28]^ and cases of ODPM with macular detachment, where no inner retinal schisis-like cavity was found.^[29]^ Todorich et al reported a case of ODPM with a direct connection of SRF to ODP, as detected on spectral-domain (SD)-OCT scans.^[30]^ Using high-resolution OCT, Imamura et al showed that fluid could move straight from the ODP to multiple retinal layers, including the sub-ILM space, ganglion cell layer, inner and outer nuclear layers, and subretinal space.^[28]^ The outer nuclear layer is usually affected – as seen in our cases; one interpretation could be that in the majority of cases, the inner retina and/or subretinal space are involved secondarily to an initial passage of fluid through the outer retina.^[31]^ Intriguingly, Skaat et al described two distinct OCT patterns in their cases: (i) A predominant serous detachment pattern, with no to minimal schisis-changes of the photoreceptor layer in pediatric patients (mean age: 9 years old). (ii) A multilayer schisis pattern in older adults (mean age: 31.7 years old).^[32]^ The former pattern in younger patients has been reported by others as well, but it was not confined to the pediatric population.^[33]^ Although direct SRF conduit from ODP is rare,^[1]^ perhaps when present, it allows fluid to pass beneath the retina much sooner than expected for conversion of asymptomatic ODP to ODPM in early adulthood. ODPM does occur unpredictably; however, blunt ocular and head trauma has been suggested as a potential trigger, especially in pediatric-onset cases. Rii et al have attributed this to the severe hyaloid face adhesion in pediatric patients exerting an anteroposterior tractional pull on the macula following trauma.^[34]^ Another explanation could be the sudden rise in ICP after trauma, forcing CSF into/under the retina.^[3]^

Nevertheless, both vitreous and CSF could serve as the pathogenic source of the accumulated fluid, passing into the retina through multiple layers, most commonly the outer nuclear layer.^[11]^ Moreover, both vitreoretinal traction and ICP pressure fluctuation play a role in the pathogenesis of the disease, either being prominent at certain ages and/or under different circumstances. We also suggest that a direct subretinal connection with the ODP cavity together with a traumatic experience may act as risk factors for accelerated progression toward ODPM in cases with asymptomatic ODP.

### Surgical Techniques in the Literature

As previously mentioned, tractional forces over the ONH allow for fluid entrance through the ODP, and the traction exerted upon the peripapillary and macular area could promote schisis separation of retinal layers, facilitating fluid migration into the retina. Therefore, complete vitrectomy with PVD induction is the mainstay of ODPM treatment.^[3,27]^ Hirakata et al showed that although, it took up to a year for the attachment to occur, the complete retinal attachment in seven of the eight eyes were achieved with isolated PPV and PVD induction.^[35]^ An alternative surgical approach is placing scleral buckles between the optic disc and macula, with a similar success rate of 85% as PPV and PVD induction. Besides alleviating vitreoretinal traction, scleral buckling is suggested to obstruct the fluid passage from ODP.^[36]^ However, this tricky technique is rarely applied in the management of ODPM.^[4]^

ILM peeling, gas tamponade, and juxtapapillary endolaser photocoagulation are commonly utilized adjunctive procedures alongside PPV. The rationale behind these procedures and their additional benefit to vitrectomy is still controversial. Pneumatic retinopexy alleviates vitreomacular traction by inducing PVD and displacing accumulated fluid;^[37]^ however, when applied without PPV, it has a temporary effect^[38]^ and a retinal reattachment rate of 50% with a mean number of 1.8 injections.^[39]^ Laser application is tentatively proposed to seal the route of the ODP to the fovea, but it has a very low success rate,^[40]^ probably because choroid and deep retinal layers absorb much of the laser energy. It also bears the risk of causing significant visual field defects.^[41]^ While re-intervention was needed in 40% of the cases, combined gas tamponade and laser application had a 75% success rate.^[42]^ After unsuccessful photocoagulation or pneumatic retinopexy, PPV and fluid air exchange, with or without laser treatment, have yielded an 88% reattachment rate.^[6]^ More recently, ILM peeling has been suggested to ensure the removal of all anteroposterior and surface-parallel traction forces from the retina.^[43]^ Three multicenter studies evaluated the surgical success rates gained with PPV and PVD induction +/– ILM peeling, gas tamponade, and juxtapapillary endolaser and evaluated the extra therapeutic gain associated with each of these adjunctive treatments^[1,15,44]^ They showed an overall 75–86% retinal attachment success rate, but no significant improvement gained with ILM peeling or temporal laser application was found; only gas tamponade showed additional efficiency in the study by Avci et al;^[44]^ however, all these studies suffer from small sample sizes and heterogeneities in the surgical procedures employed.^[1]^

Marticorena et al reported a case successfully treated with peeling of ILM after an initial failure of PVD and laser application^[45]^ PVD induction, gas tamponade, and ILM peeling resulted in favorable outcomes in three ODPM cases within a few months, as described by Georgalas et al.^[46]^ In a retrospective analysis of five patients who underwent PPV plus gas injection with or without ILM peeling, Skaat et al reported complete SRF resolution in the former group whereas macular detachment persisted in patients for whom ILM peeling was not performed.^[32]^ Even considering the different clinical and morphological characteristics of the two groups, it could still be inferred that ILM peeling is a critical surgical maneuver to alleviate maculopathy. Despite the formation of macular holes in 57% of the eyes operated, Shukla et al achieved excellent results with vitrectomy, ILM peeling, and gas tamponing in seven cases. Three out of four holes were closed spontaneously, and the final visual outcome was unaffected by macular hole development during the recovery course.^[43]^ Although fovea-sparing ILM peeling has been suggested to minimize the risk of macular hole formation,^[47]^ leaving a central island of ILM on the macula showed no protection against macular hole formation in one out of two eyes that underwent the procedure.^[43]^ Moreover, full-thickness macular hole formation has been reported following PVD, gas tamponade, and laser photocoagulation without ILM peeling;^[48]^ as Shukla et al proposed, most of the risk could be attributed to removing the strongly adherent posterior hyaloid face.^[43]^

Spaide et al introduced another adjunctive maneuver beside PPV, that is, partial-thickness fenestration of the retina, radial to ONH. It is proposed to allow fluid redirection toward the vitreous cavity instead of the intra-/sub-retinal layers.^[49]^ They later achieved 94% foveal fluid resolution with this technique.^[50]^ However, a previous attempt to create a fenestration connected to schisis cavities by Slocumb and Johnson resulted in persistent macular detachment due to premature closure of the fenestration soon after surgery.^[51]^ Moreover, this technique will not be effective in the presence of a direct conduit beneath the retina.

Sealing the congenital pit with platelet-rich plasma (PRP) or fibrin glue has shown promising results and seems to shorten the long duration of restoration following surgery.^[30,52]^ However, the plugs are temporary and cannot be regarded as a permanent solution. The long-term safety of PRP is unknown as it can theoretically trigger proliferative vitreoretinopathy.^[30]^ The use of fibrin glue carries the risk of allergic reactions and microbial transmission.^[53]^

Insertion of an inverted ILM flap over the ODP has recently been suggested; the flap could act as a physiologic physical barrier against vitreous and oil migration and induce gliosis and cell proliferation within the ODP cavity.^[54]^ Two months after complete PVD induction, ILM peeling, reverse ILM flap insertion, and gas tamponing, pit remodeling and partial closure were observed. We did not detect any macular hole formation in the three operated eyes, which was consistent with previous case reports using this technique.^[55,56]^ However, a limitation of our study should be taken into account in this regard, that is, the follow-up periods were relatively short and heterogeneous in length. Nevertheless, following a similar rationale to that of the ILM flap procedure, autologous scleral flap transplantation has also been proposed^[57]^ but has an inherent risk of optic nerve damage.^[58]^ Babu et al retrospectively compared the treatment outcomes associated with PPV+ILM peeling alone (group 1, *n* = 8), +ILM flap (group 2, *n* = 7), or +scleral flap (group 3, *n* = 8); covering of the ODP with either flap resulted in better anatomical outcomes than PPV+ILM peeling alone, and no significant difference was observed between groups 2 and 3 in terms of final anatomical results (anatomical success rates: 25% in group 1, 85.7% in group 2, and 87.5% in group 3; *P* = 0.019).^[59]^ Results from a preliminary prospective study of nine eyes undergoing PPV + translocation of an ILM flap/transplantation of an inverted ILM flap were also indicative of favorable anatomical and functional outcomes (mean pre- and postoperative CMTs: 723.4 and 398.1 microns; reattachment rate at the last follow-up: 56% [5/9]; the mean BCVA improvement corresponded to three lines of visual improvement). Treatment outcomes were comparable with either method of ILM flap covering over the ODP.^[60]^

In conclusion, surgical intervention is crucial in the management of OPDM. In addition to removing the traction over the ODP, the elevating stress over the retina and the risk of schisis formation should be minimized through PVD induction and ILM peeling, allowing retinal layers to reattach over time. However, in the presence of communicating fluid sources, the restoration process is generally slow because intermittent pressure gradients caused by fluctuations in CSF pressure could still push fluid into the retina. More recently, covering the congenital pit with an ILM flap was suggested. In our experience, the inverted ILM flap procedure is efficacious and safe in the management of ODPM. This procedure resulted in substantial functional and anatomical restoration in our three cases; eyes remained complication-free during the 7–16 weeks of follow-up. This emerging surgical technique invokes cellular proliferation and tissue construction inside the ODP and closes the communicating cavity, substantially facilitating remission. Gas tamponade may further facilitate fluid egression from the retina and keep ILM over the ODP long enough to induce and maintain cellular growth and tissue remodeling inside the pit.

##  Ethical Considerations

The protocol of the present study was approved by the local ethics committee; the study adhered to the principles of the Helsinki declaration. Informed consent was obtained from all participants.

After briefing participants on the content prepared for publication, signed informed consent letters were obtained.

##  Declaration of Patients Consent 

The authors certify that they have obtained all appropriate patients consent forms. In the form the patients have given their consent for his images and other clinical information to be reported in the journal. The patients understand that their names and initials will not be published and due efforts will be made to conceal their identities, but anonymity cannot be guaranteed.

##  Financial Support and Sponsorship

This study received no funding.

##  Conflicts of Interest

The authors declare that they have no significant competing financial, professional, or personal interests that might have influenced the performance or presentation of the work described in this manuscript.
