# Erratum: Inverted ILM Flap Technique in Optic Disc Pit Maculopathy

**DOI:** 10.18502/jovr.v18i4.14561

**Published:** 2023-11-30

**Authors:** Ali Tavallali, Yasaman Sadeghi, Seyed-Hossein Abtahi, Hosein Nouri, Sanam Samadikhadem, Mitra Rezaei, Mehdi Mazloumi

**Affiliations:** ^1^Tavallali Eye Infirmary, Shiraz, Iran; ^2^Bloomberg School of Public Health, Johns Hopkins University, Baltimore, MD, USA; ^3^Ophthalmic Research Center, Research Institute for Ophthalmology and Vision Science, Shahid Beheshti University of Medical Sciences, Tehran, Iran; ^4^Labbafinejad Medical Center, Shahid Beheshti University of Medical Sciences, Tehran, Iran; ^5^School of Medicine, Isfahan University of Medical Sciences, Isfahan, Iran; ^6^Department of Ophthalmology, Imam Hossein Medical Center, Shahid Beheshti University of Medical Sciences, Tehran, Iran; ^7^School of Medicine, Shahid Beheshti University of Medical Sciences, Tehran, Iran; ^8^Eye Research Center, Rasoul Akram Hospital, Iran University of Medical Sciences, Tehran, Iran

In the article titled “Inverted ILM Flap Technique in Optic Disc Pit Maculopathy” published on pages 320–329, Volume 18, Issue 2 of the Journal of Ophthalmic and Vision Research,^[1]^ the degree of the first author “Ali Tavallali” is “MD, FASRS” and his affiliation is “Tavallali Eye Infirmary, Shiraz, Iran”. The online version of the article has therefore been updated on October, 2023 and can be accessible from https://doi.org/10.18502/jovr.v18i2.13189.
